# Effects of Motorcycle Noise on Annoyance—A Cross-Sectional Study in the Alps

**DOI:** 10.3390/ijerph17051580

**Published:** 2020-02-29

**Authors:** Christoph Lechner, David Schnaiter, Uwe Siebert, Stephan Böse-O’Reilly

**Affiliations:** 1 Institute for Public Health, Medical Decision Making and Health Technology Assessment, Department of Public Health, Health Services Research and Health Technology Assessment, UMIT—University for Health Sciences, Medical Informatics and Technology, 6060 Hall in Tirol, Austria; 2 Office of the Tyrolean Regional Government, Department for Emission, Safety and Sites, 6020 Innsbruck, Austria; 3 Independent Researcher Environment and Public Health, 6020 Innsbruck, Austria; 4 Institute and Clinic for Occupational, Social and Environmental Medicine, University Hospital, LMU Munich, 80336 Munich, Germany; 5 University Children’s Hospital Regensburg (KUNO-Clinics), University of Regensburg, Clinic St. Hedwig, 93049 Regensburg, Germany

**Keywords:** annoyance, road traffic noise, motorbike noise, motorcycle noise, exposure-response functions, non-acoustic factors, background noise

## Abstract

Motorcycle noise is an increasing noise problem, especially in Alpine valleys with winding roads and low environmental noise. The annoyance response to motorcycle engine noise is extraordinarily high in comparison to other traffic noise and cannot be explained by standard noise assessment curves. Therefore, the Tyrolean state government decided to initiate a multi-purpose study. Exposures were calculated based on sound-measurements taken across the entire district of Reutte in the western part of the State of Tyrol and a telephone survey (*n* = 545) was conducted with regional participants. The influence of demographic characteristics; sensitivity to noise; attitudes towards motorcycles and background noise on the annoyance was examined using bivariate analyses. In addition; exposure-response curves and their 95% confidence intervals with cut-off points of 60% and 72% for “highly annoyed” were created. The exposure annoyance response curves for motorcycle noise show a shift of more than 30 dB in annoyance reaction compared to other road traffic noise. The annoyance response to motorcycle noise in this Alpine region is concentrated on summer Sundays and Saturdays and is independent of the background exposure caused by other road traffic

## 1. Introduction

Noise is a relevant problem to the public and to individuals. Noise pollution in Europe is regarded as a serious environmental problem [1], in Austria it is even the prior-ranked problem concerning environment conditions [2]. Noise has important health-related consequences such as ischemic heart disease, cognitive impairment in children, sleep disturbance, tinnitus and annoyance [3]. The effects of annoyance are particularly high in connection with traffic noise [4]. The European Directive 2002/49/EC [5] recommends the use of exposure-response relations between the noise indicator L_den_ and noise annoyance as determined by means of field surveys. The noise indicator L_den_ for overall annoyance is an A-weighted long-term average sound level at which the evening receives an addition of 5 dB, and for the night-time of 10 dB. The most recent review on environmental noise and annoyance presents such a relation for road traffic noise [6]. The annoyance models are based on typical percentages of light and heavy vehicles in the traffic flow. The reports do not mention the analysis of situations where a substantial part of the total noise results from two-wheelers [6]. There is a research gap in assessment and explanation of the annoyance reaction on motorcycle noise

The Tyrolean Außerfern (political district Reutte), with approximately 35,000 inhabitants, is a very popular area for motorcycle tourism due to the beautiful landscape and winding roads. The residents have been sending complaints about the noise of motorcycles to the responsible authorities and politicians for years. These conflicted interests have to be resolved by the authorities whereas traffic limiting measures based on Section 43 (1) or (2) of the Austrian road traffic act [7] can only be taken to protect the public or the environment from foreseeable hazards or unreasonable nuisances—in this case extraordinary noise, odor or pollutants. Every permanent or temporary traffic restriction or traffic prohibiting measure for all or for certain types of vehicles (or for vehicles with certain cargoes) has to be proven to be a necessary measure. It cannot be introduced as a purely political decision [7].

Previous investigations on the southern side of the Hahntennjoch route [8,9] showed that there was no difference in noise levels between motorcycles and cars in the low speed range of 30 km/h as expressed according to the A-weighted standard [10] in technical acoustics. A discrimination of motorcycles in compliance with the prescribed speeds was technically not justified. Nevertheless, the reality appears to be very different when looking into the real driving situation out on the streets and arguments are being sought to either justify or dispel restrictions on motorcycles. In Switzerland, a survey on motorcycle noise was conducted amongst visitors of a wildlife park [11]. For the purpose of motorcycle noise assessment, a separate sound prediction model was developed [12]. The results of these studies show high levels of annoyance, although they were not transferable to the resident population. Additionally, different studies in inner-city areas have also indicated a special annoyance situation caused by mopeds and motorbikes [13,14,15]. Therefore, there is a research gap regarding the evidence for the effect of motorcycle noise on the extent of annoyance of inhabitants in rural areas. In the most recent micro-census report [2], it is shown that 9% of Austrians above 15 of age listed motorcycles as dominant noise generators, while passenger cars accounted for 16.4%. The vehicle registration statistics [16] show a completely different picture as 72.2% of all motor vehicles in Austria are passenger cars whereas only 7.7% are motorcycles. Hence our overall aim was to determine the magnitude and to identify the reasons for the strikingly different annoyance reactions observable between cars and motorcycles.

To answer these questions, we investigated the actual burden of motorcycle noise for the affected residents in the district of Reutte and their annoyance response to it. In addition, we compared the exposure and response from motorcycle noise to those from the remaining traffic, that is cars and heavy vehicles, and we investigated the effect of motorcycle noise on quality of life. We also explored the reasons for the annoyance response to motorcycle noise.

## 2. Materials and Methods

### 2.1. Design and Sample

As the motorcycle noise problem reached a peak in spring 2018, the study was carried out in summer 2018 by the Office of the Tyrolean Regional Government, Department for Emission, Safety and Sites. The first author acted as project manager, the second author as contractor. A cross-sectional study design was implemented sampling the whole district of Reutte (at least 500 telephone interviews). The sampling was planned according to three basic principles, that is; (1) demographic representativeness, (2) distribution of inhabitants within the communities, and (3) classification according to the increase of traffic noise due to presence of motorcycles in three groups depending if the noise level increase caused by motorcycle noise is little, medium or strong.

The study area (political district Reutte), also called “Außerfern”, has an area of 1236 km² and had a population of 32,532 registered main residents living in 37 communities in 2018. Reutte is the district capital and by far the largest municipality in the district with 6638 inhabitants at the time of writing. The population density is very low with 26 inhabitants per square kilometer. In the population of Reutte 50.5% are female, 31% are 20 to 39 years old, 38% between 40 and 59 years and 31% older than 31%. 30% has a primary, 62 a secondary and 8% a tertiary level of education. The proportion of foreign national is 19%. The labor force participation rate in the population aged between 15 and 64 is 70%.

The survey was conducted using Computer Assisted Telephone Interviews (CATI) and operated by the market research company Institut für Marktforschung und Datenanalyse (Institute for Market Research and Data Analysis, IMAD, Innsbruck, Austria) on behalf of the contractor. We specified the random sample selection in detail as follows: clusters given by age, gender, number of inhabitants within the municipalities and noise exposure groups according to the increase of total traffic noise due to motorcycle noise. As parameter to describe the increase of the remaining road traffic noise due to presence of motorcycles, the equivalent continuous level on summer Sundays during daytime between 6 a.m. and 7 p.m. (ΔL_SS_) in dB was chosen. The exposure was calculated as an average over the months of June to September based on traffic counts provided by the Traffic Planning Department of the State of Tyrol. This index is defined as equivalent continuous level of the whole traffic minus the equivalent continuous level of the traffic without motorcycles in the defined timeframe. The noise exposure groups were ranked as follows: increase by motorcycle noise “little” for ΔL_SS_ lower than 0.5 dB, a medium for ΔL_SS_ between 0.5 and 1.0 dB and strong for ΔL_SS_ greater than 1.0 dB. Between November 19th and December 17th, 2019, a total number of 571 full telephone interviews were completed by 14 trained interviewers. The database was handled and anonymized by the survey institute in compliance with all quality assurance and data protection requirements according to the European General Data Protection Regulation [17] and Austrian legal regulations and prepared for further statistical evaluation.

### 2.2. Means and Instruments

The exposure to noise was predicted according to the Environmental Noise Directive [5] with the Austrian interim methods [18]. We created a three-dimensional model of the whole district of Reutte. The terrain model is based on a 3D point cloud resulting from the laser scan localization which had been prepared according to defined requirements for the present project area. The laser scanning was carried out by the State of Tyrol in the period 2012 to 2015 [19]. The maximum vertical distance between two height points was chosen to be 0.1 m. In addition, a maximum horizontal distance of 10 m was set.

In the evaluation of the buildings, the building polygon determined by the laser scan positioning was reduced in circumference by one meter to eliminate any bottom stitch of the laser scan location. This would be significant for the calculation of the building height impact. To obtain the height of the building, we calculated the arithmetic mean of minimum and maximum values within the reduced polygon. This fulfils the requirements of the Austrian guidance recommendations [20].

**Figure 1:**
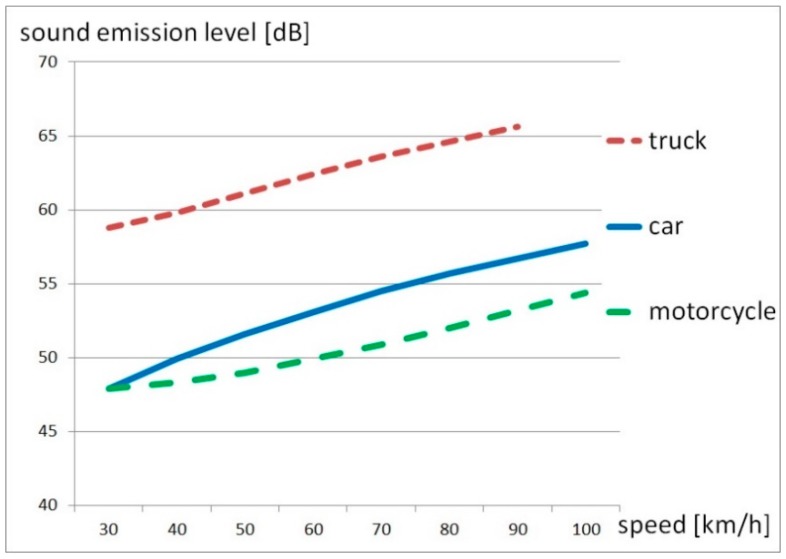
Comparison of the sound emission level according CNOSSOS-EU [21] between motorcycles, cars and trucks on concrete asphalt.

**Figure 2:**
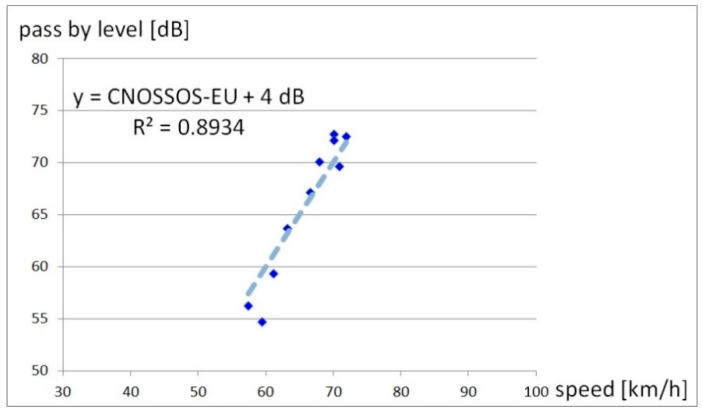
Adjusting the motorcycle emission model; mean values of adjusted measurements and linear regression line for adaption of motorcycle emission values by +4 dB.

We used the Austrian standard method RVS 04.02.11 [18] for road traffic noise to predict the noise exposure in the reference year 2017. Following this method, motorcycles are to be predicted like single heavy vehicles. To test this assumption, we conducted several traffic noise measurements on 10 dedicated points on different days of the week and investigated single pass-by-levels of motorcycles and other vehicles. These measurement values were adjusted for equal distance and compared with the sound emission level of CNOSSOS-EU [21]. This level depends on the road layer, speed, temperature, humidity and type of vehicle type. The level sums up propulsion noise and rolling noise that is neglected in motorcycle noise emission. As shown in Figure 1, there is a relevant deviation between the noise emission values of cars, trucks and motorcycles on the Tyrolean standard layer asphalt concrete:

Since we observed in our analysis that motorcycles are certainly not less noisy than cars or trucks, a linear regression curve fit was conducted as shown in Figure 2.

After this regression, the emission values were adapted for the overall calculation with a correction term of +4 dB to minimize deviation.

The calculations considered reflections of the sound path on objects of 1st order. Receiver points were assigned to the building façades at a height of 4 m. As a result, the loudest façade levels for the different noise contributors were associated to each address and used to classify inhabitants and dwellings. The grid width for noise mapping was chosen to be 10 m × 10 m. Input data were provided from 2017. The velocities of each vehicle category were modelled according to the legal speed limits. Interactive noise maps of the exposure, as well as the differences in levels due to motorcycle traffic, are available in high resolution at https://tirol.gv.at/motorradlaerm-reutte.

On the one hand, the questionnaire was based on standard noise assessment items and scales, while, on the other hand, it was specifically developed and oriented towards the current state of knowledge of noise impact research.

The questions about annoyance caused by traffic noise and the corresponding rating scales (5-point scale) were based on the recommendations of the “International Commission on Biological Effects of Noise (ICBEN)” [22]. Proven items from comparable studies (e.g., NORAH [23], LEF-K on noise sensitivity [24], EU-SILC 2015 [25], micro-census “Environmental Conditions” and “Health” [2], other surveys in Tyrol [26,27] were also considered. Most of the socio-demographic questions were accepted in a standardized way.

The study was reported to the university research committee for scientific and ethical questions (RCSEQ) at UMIT. The RCSEQ replied that since no personal sensitive data is processed in this research project (retrospective analysis of anonymized data), a RCSEQ vote is not required. The scientific work has been documented as an RCSEQ report. Data protection is given by the Government of the State of Tyrol, fulfilling all legal requirements.

For our study, the questionnaire had to focus on to a considerable extent on the specific motorcycle issues, and thus needed to be developed by the authors as it could not be based directly on existing modules. The core areas in the questionnaire were:

- Socio-demographic (age, gender, level of education, etc.)
- Mobility (use of public transport, car and motorcycle)
- Living conditions and quality of life
- Subjective assessment of self-reported health status
- Subjective assessment of self-reported quality of life
- Subjective assessment of self-reported noise sensitivity
- Changes in road traffic noise in recent years
- Annoyance/disturbance through noise from motorcycles and other vehicles
- Dealing with motorcycle noise (effect of the PR-action “Please drive quietly”, extent of interference in comparison, disturbance depending on season/weekday/time of day, disruption of activities, peculiarities of the disturbance caused by motorcycles and motorcycle noise, own contribution to overall noise, approval of further measures for noise reduction, opinion on motorcycle traffic, etc.)

The source specific annoyance was determined in accordance with the international standardized format of the ISO/TS 15666 [28]. The 5-point scale of annoyance was used, ranging from (1) not at all; (2) slightly; (3) moderately; (4) very to (5) extremely.

### 2.3. Statistical Analyses

The evaluation of the results was carried out by applying descriptive statistics and bivariate analyses. This was performed by group comparisons. Non-parametric methods such as the Mann-Whitney U test and the Kruskal-Wallis test were used as test methods after analysing the distribution form.

In the present analyses, a comparison of the grouped response values 4 and 5 (e.g., %HA, percentage highly annoyed) and 1 to 3 (e.g., %NHA, percentage not highly annoyed) were used as cut-off points for the 5-point response scales according to the ICBEN recommendations [22]. Many recent studies [6] use a cut-off point at 72% according the answer categories of 8, 9 and 10 on the 11-point scale as “highly annoyed”. To provide a readily comparison with exposure-response functions from these studies a transformation of the 5-point scale was performed by weighing the annoyance responses of category 4 (very) with a weight of 0.4 in line with [29,30]. To create an exposure-response function by a binary logistic regression a weight of 0.4 was achieved by randomly distribution “highly annoyed” and “not highly annoyed” in answer category 4 with a ratio of 4/6. The two different percentages for highly annoyed are indicated by HA_60_ and HA_72_ depending on the cut-off points.

To categorize the general attitude towards motorcycles, a factor analysis was conducted based on six items asking for approval or rejection of measures to restrict motorcycle traffic. The categorized factor analysis was rated by Kaiser-Meyer-Olkin’s measure of sampling adequacy.

To describe the independent influence of sound exposure of different vehicle types on noise annoyance multivariate logistic regression analysis was used. For this purpose, the cut-off point of 60% for “highly annoyed” HA_60_ was chosen. The covariates for the logistic regression were determined by applying univariate analyses. The purposeful selection process began with a univariate variable screening for each variable. Any variable having a significant univariate test result at some arbitrary level was selected as a candidate for the multivariate analysis. We based our analyses on the Wald test from the logistic regression and a *p*-value cut-off point of 0.2. Significant results at the 0.1 level were incorporated into the multivariate regression model. Consequently, the model was iteratively reduced as before but only for the variables that were additionally added [31]. The influence of traffic noise caused by cars and heavy vehicles on the annoyance of motorcycle noise was tested by a logistic regression analysis in a generalized linear model (GLM). The normal distributions of the dependent variables were tested using the Kolmogorov-Smirnov-test.

The influence of motorcycle noise on quality of life was described by noise exposure grouped in 5 dB steps. Exposure-response curves were calculated for the noise indicator L_d,SS_. This indicator describes the equivalent continuous level on summer Sundays during daytime between 6 a.m. and 7 p.m. as an average over Sundays from June to September. Source-specific exposure-response-curves on the percentage of highly annoyed people were calculated using binary logistic regression analysis in a GLM.

All analyses were performed with the statistical analysis software package SPSS 23 (IBM, Armonk, NY, USA).

## 3. Results

### 3.1. Survey Response Rate and Reliability

The response rate is defined as the ratio of successful (complete) interviews to potential interview persons encountered who fit in the cluster sample. As a result of several applied procedures, especially the intense field preparation for the survey through different media channels, a response rate of 74.8% was achieved (see Table 1):

The effectively achieved response rate ensures a high representativeness of the results minimizes system inherent bias (self-selection, sampling error, lack of representativeness, etc.) and is exceptionally high compared to recent international studies, especially for a telephone survey.

Statistical reliability tests conducted as shown in Table 2 below:

### 3.2. Descriptive Statistics

Five hundred and seventy one (571) persons were interviewed in the survey. The descriptive statistics of the full dataset have been published earlier [27]. Of all interviewed participants, 26 were excluded in this study because the noise exposure data could not be assessed accurately. Briefly, the main characteristics of the sample are summarised in Table 3.

Participants were asked about the extent of personal noise annoyance caused by different road users. Table 4 below shows the percentages of highly annoyed people, mean values, standard deviations and test statistics according to Kolmogorov-Smirnov.

More than 80% of the participants felt particularly disturbed by road noise with nearly 45% at a cut-off point of 60% respectively 31% at a cut-off point of 72% of them being highly annoyed solely by motorcycle noise alone. Between 8% and 14% of the respondents sensed disturbances from motorcycle noise while performing activities within their house or dwelling (watching TV, listening to music, conversing, making a phone call, sleeping, receiving visits and socializing, reading or relaxing). The disturbance caused by motorcycle noise thus relates to a relatively small proportion with indoor activities. Conversely, 67% felt annoyed or disturbed by the noise of motorcycles when performing outdoor activities, for example in their own garden. Some special characteristics of the disturbance by motorcycles were answered as follows in Table 5.

### 3.3. Motorcycle Noise and Quality of Life

The impact of different noise sources on the participant’s self-reported quality of life was assessed by mean values grouped in noise bands in 5 dB steps. The overall rating of the respondents was 48.3% “very good”, 41.2% “good”, 8.6% “moderate”, 1.2% “bad” and 0.7% very bad. By comparing the correlation of quality of life and noise annoyance from motorcycles and other vehicles, a significant difference between these two groups can be observed (*p* = 0.000).

**Figure 3:**
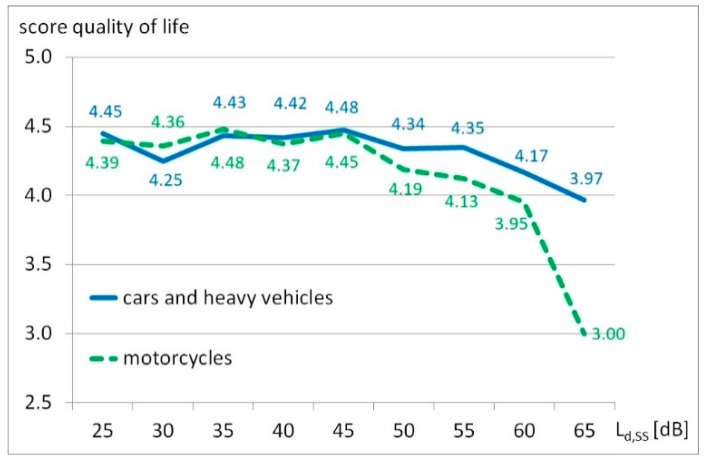
Mean values for quality of life by noise exposure groups expressed as equivalent continuous level during daytime on summer Sundays L_d,SS_ in 5 dB steps from motorcycles and cars and heavy vehicles.

Figure 3 reproduces the mean values for quality of life by noise exposure groups in 5 dB steps from motorcycles and other vehicles.

### 3.4. Purposeful Selection of Covariates in the Logistic Regression

As a result of a factor analysis, a variable for the general attitude towards motorcycles was created. The result of the Kaiser-Meyer-Olkin measure of sampling is 0.7 which is rated as moderate. The influence of some variables on noise annoyance expressed as highly annoyed (%HA_60_) is shown in Table 6.

The final model only included self-reported noise sensitivity and attitude to motorcycles. The typical epidemiological characteristics gender and age as covariates did not meet the inclusion criteria.

### 3.5. Influence of Road Traffic Noise Sources on Annoyance by Motorcycle Noise

Binary logistic regressions in a GLM were used to analyse if there is an influence of background noise caused by 4-wheelers on the annoyance response to motorcycle noise. According to the results of the bivariate variable screening, self-reported noise sensitivity and attitude to motorcycles were included in the multivariate model (see Table 7).

### 3.6. Source-specific Exposure-Response Relationship for Transportation Noise Annoyance

**Figure 4:**
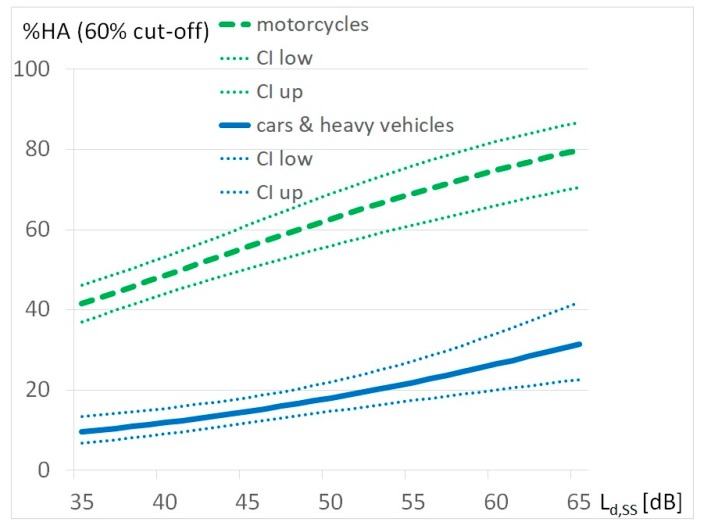
Exposure-response relationship %HA_60_ for motorcycles and other vehicles (cars and heavy vehicles) adjusted by noise sensitivity and attitude to motorcycles by noise exposure during daytime on summer Sundays L_d,SS_.

**Figure 5:**
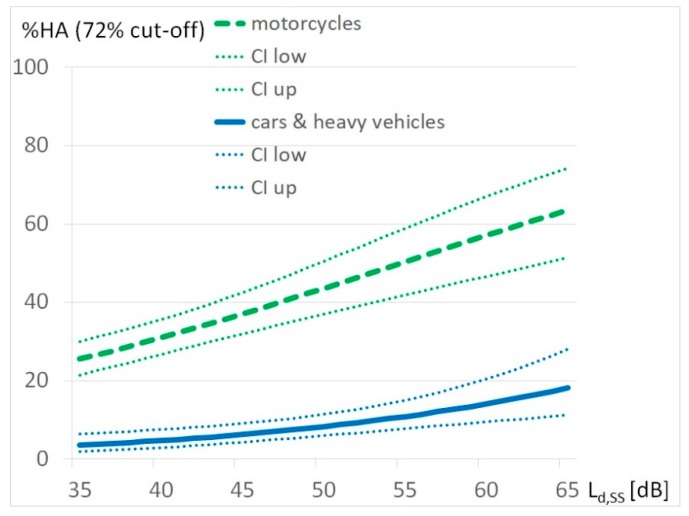
Exposure-response relationship % HA_72_ for motorcycles and other vehicles (cars and heavy vehicles) adjusted by noise sensitivity and attitude to motorcycles by noise exposure during daytime on summer Sundays L_d,SS_.

A logistic regression analysis was conducted to assess the exposure-response-relationship of single source-specific noise by motorcycles and other vehicles—cars and heavy vehicles—and noise annoyance. The chosen period for this comparison is the average summer Sunday during daytime (the point of time with highest motorcycle noise burden). The exposure to motorcycle noise has a mean value M = 36.8 dB, a maximum Max = 63 dB and a standard deviation Std = 11.4 dB. The exposures due to other vehicles (cars and heavy vehicles) are M = 43.9 dB, Max = 70.3 and Std = 11.4 dB. Source-specific exposure-response relationships for “highly annoyed” by noise exposure on summer Sundays for the cut-off point of 60% are shown in Figure 4, for the cut-off point of 72% in Figure 5 below:

## 4. Discussion

This study was carried out in the Tyrolean district of Reutte in 2018, following frequent complaints about motorcycle noise. The extent of noise pollution from motorcycle traffic and other road users was surveyed. The extent of the nuisance was recorded with these exposure values and via a telephone survey. 44.8% of the respondents claimed to be highly annoyed at a cut-off point at 60%, 30.8% at a cut-off point at 72% while the mean exposure value was at 34 dB on summer Sundays and 33 dB as an annual average. From this, exposure-effect relationships were performed. The exposure annoyance response curve created using binary logistic regression for motorcycle noise shows a shift of more than 30 dB in annoyance reaction compared to other road traffic noise.

Previous studies investigated different effects on annoyance by powered two-wheelers. While a study from the Netherlands had mopeds in focus [15], others had different settings [13,14] and a study from Switzerland was based on different respondents [11]. A lack of research in alpine and rural settings focusing on the annoyance reaction due to heavy motorcycles is addressed by this study.

There is a common tendency in all these studies to assume that moped and motorcycle noise is more annoying than noise from other vehicles. The result of our study, presented as an exposure-response curve in Figure 4, is in line with these findings but shows a much higher penalty for motorcycles than estimated by other studies, which was generally estimated at 5 dB [14,15].

An accurate determination of noise exposure is the basis for valid exposure-response curves. Underestimating the exposure leads to overestimated effects. To avoid such an error, sound measurements were conducted in our study. These measurements have shown that the standard prediction model does not accurately depict the real situation of motorcycle noise. There are multiple reasons for this. For one thing, the acceleration processes at the limits of different speed restrictions are not adequately mapped in the calculation model. Another reason could be the emission approach itself. In Austria, vehicle-specific emission input parameters were not changed during the implementation of the CNOSSOS-EU method [21]. The adaptation was carried out exclusively via the road surface related parameters resulting in an adjustment of 0.5 dB for the A-weighted level for each speed level [32]. In the meantime, several suggestions have been made concerning the emission values for the CNOSSOS-EU method [33], as the current ones are underestimating. According to these findings the Austrian Ministry for Transport, Innovation and Technology decided to withdraw the prediction method [34] and the EC proposed an amendment to the commission directive [21].

For motorcycles, there has been no adaptation in the prediction method so far because only propulsion noise, and no rolling noise, was considered. So, all factors were adjusted in the Austrian national method, except for motorcycles [34]. This partially explains the deviations found in this study. These deviations could be compensated for with a correction value of 4 dB and real exposure to motorcycle noise could be correlated with the survey results.

Our study also investigated in which time frames exposures (sound pressure level) and responses (annoyance score) correlate best. The sound exposure was determined separately for motorcycles, cars and heavy vehicles and mapped for different time frames. In a logistic regression analysis, it was examined which time frame matches the annoyance reactions best. We found significant results for Sundays and Saturdays in summer (the highest amount of motorcycle excursion traffic). Notably, the results during summer weekdays and whole summer weeks, as well as for overall exposure during the year, were of no significance. In the evaluation of motorcycle noise, attention should be paid to this circumstance.

According to Austrian legislation, annoyance must be assessed by the change of the local ambient noise caused by a specific noise source. It is assumed that the higher the level increase caused by an additional source is, the more annoying the noise of that specific source is. Table 7 provides information about the influence of sound levels from motorcycles and other vehicles and from self-reported sensitivity to noise and attitude to motorcycles. The investigated periods include Sundays, Saturdays, weekdays and the whole week, both as a yearly average and as an average during the summer months. The same results occur in all cases. Annoyance reactions to motorcycle noise are not significantly influenced by the sound levels of other vehicles. No significant influence of the noise from other vehicles on annoyance from motorcycles shows up in any of these periods. There is no observable influence of the background noise from cars and heavy vehicles. The response to motorcycle noise is independent and it is correlated to summer Sundays and Saturdays because of the increased amount of motorcycle excursion traffic. In contradiction to the Austrian legal status, no protective effect of ambient noise from other vehicles to motorcycle noise is visible.

As already shown in the previous published overall noise analysis of the total noise investigation in Innsbruck [27], quality of life is a very sensitive and informative sum score for noise pollution. The most affected periods by motorcycle noise (in summer on Sundays during the day) show minor differences in the perception of the sources of noise in combination with the assessment of one’s own quality of life. The same is valid for the quality assessment of personal living conditions. While the “threshold values” of traffic noise exposure, based on the level at which the estimate of self-reported quality of life begins to decrease, is around 55 dB in the case of cara and heavy vehicle traffic noise, it can be already observed at levels of 45 dB for motorcycle noise.

This relationship between personal quality of life and annoyance due to road traffic noise is also statistically significant. In particular, those “highly annoyed” by traffic noise as a whole estimate their personal quality of life as significantly less good than those “not highly annoyed”.

As known from other studies [13,14,15], motorcycle noise shows significantly higher annoyance reactions than other vehicle traffic noise. It is questionable as to what extent this reaction can be attributed to the political discussion about traffic noise in the region. Further, in the vicinity of Frankfurt Airport [35] extraordinarily high annoyance reactions were described during and after its controversially discussed extension. People’s awareness of the topic is also reflected in the response rate which clearly exceeds comparable studies [23]. The politically heated mood in the Außerfern district suggests an effect overestimation. When comparing the results of exposure-response curves, it is also important to consider the timing of the study.

Table 7 proves that the average annoyance response to total road traffic noise is lower than to motorcycle traffic noise. As shown in a recent study in Tyrol [36], the total annoyance results from the components of the individual noise sources. This assumes that the total annoyance is higher than that of the individual components. An overall noise assessment model can hence not be assessed correctly from individual components of different road users.

The accurate exposure modelling is an advantage of the present study. Due to the correction of the sound emission an overestimation of the effect could be avoided. An additional strength in this study is the high response rate. The high response rate based on the intense field preparation contains also a risk of bias. To ensure a neutral report from media we provided media information with appropriate length and content that where widely used by the media. In addition, a neutral information was given by the head of the district to all communities encouraging all inhabitants to participate in the survey.

This study also has several limitations. One limitation is certainly the range of motorcycles in the area. The proportions of large-volume and frequently deep-sounding American motorcycles as well as the high-revving Japanese racing models were very low. These types of motorcycles might cause different noise reactions in other areas. In accordance with the differing acoustical characteristics of these different motorcycles it would also be relevant to investigate the impact of these characteristics’ occurrence. In the current study, only average sound pressure levels were examined. The politically heated mood in the Außerfern suggests an overestimation of the annoyance response in our study. Interviewees expect political measures may therefore have given higher values in their annoyance judgment in order to enlarge the problem dimension. This is shown by the average annoyance on total noise exposure, which is lower than the one on motorcycle noise exposure alone. It can be assumed that the interviewees have, at least partially, a differential perception of traffic noise in mind not comprising the noise of motorcycles. Therefore, an additional investigation in a region where no citizen movement against motorcycles takes place is recommended. The results of our study are only partially generalizable to other regions in Tyrol or the neighboring countries. Transferability to other regions or settings strongly depends on the comparability of the dominant motorcycle types, the exposure, the types of development and the demographic characteristics of the population.

## 5. Conclusions

The present study shows that the relation between the noise indicator L_den_ and annoyance response to motorcycle noise is substantially different compared to road traffic in general. The exposure annoyance response curve for motorcycle noise shows a shift of more than 30 dB in annoyance reaction compared to other road traffic noise. Measures against motorcycle noise would therefore have to be oriented towards temporary avoidances of motorcycle noise as a whole. A mere limitation of the number of motorcycles is not promising. For this reason, the authors recommend temporary bans on motorcycles on all heavily travelled sections in the region, for example, on Sundays during summer months. An additional step to reduce the noise burden and the annoyance would be the ban of loud motorcycles. This would correspond with the preferred measures of the population. For this purpose, options for detection and monitoring are necessary at a legal level.

## Figures and Tables

**Table 1:** Response rates in the noise clusters of strong, medium and little affected address points.

Item	Number	Percent
Conducted interviews at first contact	506	66.2%
Conducted interviews after prior appointment	66	8.6%
Refused interviews	57	7.4%
No appropriate person present	102	13.3%
Incomplete interviews	0	0.0%
No telephone contact established (no answer, wrong number)	34	4.5%
Sum of all attempted telephone calls	764	100.0%
Sum of all established telephone contacts	730	95.5%
Sum of all established telephone contacts with valid persons (age, Gender, main residence, valid noise exposure group)	628	82.2%
Sum of all successfully conducted telephone interviews	571	74.8%

**Table 2:** Reliability test results on item sets.

Reliability Statistics	No. of Items	Cronbachs Alpha	Rating
Specific annoyance	4	0.857	excellent
Disturbed time periods	3	0.666	very good
Reason of disturbance	4	0.519	good
Disturbed activities	6	0.819	excellent
Noise mitigation measures	11	0.665	very good

**Table 3:** Overview of the descriptive statistics for the main study population characteristics of the survey (*n* = 545 participants).

Characteristic		*n* (%)
Gender	female	282 (51.7)
	male	263 (48.3)
Age group	19–40 years old	151 (27.7)
	41 to 60 years old	215 (39.4)
	over 60 years old	179 (32.8)
Highest educational level so far achieved	primary level	60 (11.0)
	secondary level	401 (73.6)
	third level	84 (15.4)
Motorcyclist	motorcyclist	151 (27.7)
	not motorcyclist	394 (72.3)
Noise level increase due to motorcycles	none/little	162 (29.7)
	medium	224 (41.1)
	strong	159 (29.2)

**Table 4:** Percentages of highly annoyed people, mean values, standard deviations and test statistics according to Kolmogorov-Smirnov for different road users (*n* = 545).

Annoyance	Road Traffic Total	Cars and Heavy Vehicles	Motorcycles Only	Heavy Vehicles Only
Number HA_60_ (%)	144 (26.4)	88 (16.1)	244 (44.8)	120 (22.0)
Number HA_72_ (%)	85 (15.6)	44 (8.1)	168 (30.8)	68 (12.5)
Mean (±STD)	2.59 (±1.286)	2.29 (±1.135)	2.97 (±1.542)	2.30 (±1.300)
*p* value	0.000 ^a^	0.000 ^a^	0.000 ^a^	0.000 ^a^

Note. HA60 “highly annoyed with a cut-off value at 60%; HA72 “highly annoyed with a cut-off value at 72%; a = Lilliefors Significance Correction.

**Table 5:** Special characteristics of disturbance, preferred mitigation measures and effectiveness of action “please drive quietly”.

Item	Response	*n* (%)
Special characteristics of the disturbance caused by motorcycles:	high revolutions while accelerating	415 (76.1)
	fast and aggressive riding	363 (66.6)
	groups of motorcycles	302 (55.4)
	low frequencies “humming”	91 (16.7)
Preferred measures on motorcycles	more and strict traffic controls	454 (83.3)
	higher fines for loud motorcycles	445 (81.7)
	awareness sensitisation	437 (80.2)
	ban on loud motorcycles	424 (77.8)
	banned on specific routes	365 (67.0)
	additional speed limits	284 (52.1)
	general ban of overtaking	244 (44.8)
	toll charges for motorcycles	24 (44.0)
	more physical measures (like traffic refuges)	179 (32.8)
	banned on weekends	152 (27.9)
	general ban on driving for leisure traffic	69 (12.7)
Effect of PR-action “please drive quietly”	action unknown	49 (9.0)
	no effect	195 (35.8)
	little effect	146 (26.8)
	strong effect	26 (4.8)
	do not know	129 (23.7)

Note: No answer missing; *n* = 545.

**Table 6:** Univariate correlation of highly annoyed from 2-wheelers and 4-wheelers with other characteristics.

		Regr. Coeff.	Odds Ratio	95% CI		*p*
				Lower	Upper	
A_dich,HA,2w_	age	0.005	1.005	0.994	0.399	0.399
	age²	0.000	1.000	1.000	1.000	0.507
	gender	−0.350	0.705	0.502	0.043	0.043
	sensitivity	0.448	1.566	1.314	0.000	0.000
	attitude	−1.377	0.252	0.176	0.000	0.000
	education	−0.106	0.899	0.646	0.529	0.529
	biker	0.247	1.280	0.875	0.204	0.204
A_dich,HA,4w_	age	0.012	1.012	0.997	0.114	0.114
	age²	0.000	1.000	1.000	1.000	0.157
	gender	−0.411	0.663	0.417	0.083	0.083
	sensitivity	0.323	1.381	1.101	0.005	0.005
	attitude	−0.937	0.392	0.238	0.000	0.000
	education	0.006	1.006	0.645	0.977	0.977
	biker	0.238	1.268	0.746	0.380	0.380

Note. A_dich,HA,2w_ = percentage of highly annoyed by noise of 2-wheelers i.e., motorcycles; A_dichHA,4w_ = percentage of highly annoyed by noise of 4-wheelers i.e., cars and heavy vehicles; CI = 95% confidence interval; p = significance level.

**Table 7:** Effect of Noise level in different time frames, noise sensitivity and attitude to motorcycles on annoyance in multivariate models binary logistic regression due to the influence of road traffic background noise as *p*-values.

Parameter	B	95% CI		*p*
		Lower	Upper	
(Intercept)	2.921	1.835	4.006	0.000
L_2w_ summer Sunday	−0.075	−0.139	−0.011	0.021
L_4w_ summer Sunday	0.018	−0.045	0.081	0.576
Sensitivity	−0.440	−0.624	−0.255	0.000
Attitude	1.271	0.882	1.659	0.000
(Intercept)	2.858	1.763	3.952	0.000
L_2w_ summer Saturday	−0.075	−0.137	−0.013	0.018
L_4w_ summer Saturday	0.018	−0.043	0.079	0.559
Sensitivity	−0.437	−0.622	−0.252	0.000
Attitude	1.271	0.883	1.659	0.000
(Intercept)	3.079	1.953	4.205	0.000
L_2w_ summer weekday	−0.018	−0.074	0.037	0.521
L_4w_ summer weekday	−0.034	−0.088	0.021	0.223
Sensitivity	−0.436	−0.621	−0.250	0.000
Attitude	1.339	0.945	1.733	0.000
(Intercept)	3.012	1.879	4.145	0.000
L_2w_ summer week average	−0.031	−0.092	0.029	0.305
L_4w_ summer week average	−0.021	−0.080	0.038	0.480
Sensitivity	−0.437	−0.622	−0.251	0.000
Attitude	1.338	0.944	1.733	0.000
(Intercept)	2.837	1.691	3.984	0.000
L_2w_ entire year week average	−0.028	−0.082	0.026	0.313
L_4w_ entire year week average	−0.022	−0.076	0.032	0.423
Sensitivity	−0.431	−0.617	−0.245	0.000
Attitude	1.338	0.946	1.731	0.000

Note. L_2w_ = Sound pressure level during daytime caused by 2-wheelers i.e., motorcycles; L_4w_ = Sound pressure level during daytime caused 4-wheelers i.e., cars and heavy vehicles; B = regression coefficient; CI = 95% confidence interval; *p* = significance level.
